# Supplementary material for: Comparative Performance of Digital PCR and Real-Time RT-PCR in Respiratory Virus Diagnostics
Source: Viruses. 2025 Sep 18;17(9):1259. doi: 10.3390/v17091259 (PMC12474457; doi:10.3390/v17091259)

Comparative Performance of Digital PCR and Real-Time RT-PCR in Respiratory Virus Diagnostics

Supplemetary Materials

Supplemetary Table S1: Respiratory samples subjected to dPCR analysis, grouped according to the type of virus.

Specimens are listed in rows with virus detection status indicated in columns to facilitate identification of co-infections. Real-Time RT-PCR positivity was defined as Ct < 40.

Positive controls were not included in the dPCR workflow. This is because the study was based on retrospective analysis of anonymised clinical specimens collected during routine diagnostics. All samples included in the study were previously confirmed positive for at least one target virus using CE-IVD validated Real-Time RT-PCR assays. * Data not included in the statistical analysis

15

	Real-Time RT-PCR (Ct)						dPCR (viral copies/µl)				
Sample	Flu A_Ct	Flu B_Ct	RSV_Ct	SARS-CoV-2_Ct	NEG_Ct	dPCR dilution	Flu A_copies	Flu B_copies	RSV_copies	SARS-CoV-2_copies	NEG_copies
1	32					undiluted	9.11				
2	18					1/100	7506				
3	26					1/100	1896				
4	32					undiluted	1.14				
5	23					1/100	638440				
6	21					1/100	311890				
7	23					1/100	132040				
8	19					1/100	479010				
9	22					1/10	8344				
10	20					1/100	51040				
11	31					1/10	11.02				
12	28					1/10	561.60				
13	22					1/100	13160				
14	20					1/100	244650				
15	22					1/100	77640				
16	35					1/100	796.80				
17	32					1/10	69.70				
18	29					1/10	5.42				
19	32					1/10	9.93				
20	22					1/100	8617				
21	24					1/100	5929				
22	25					1/100	6387				
23	18					1/100	775260				
24	20					1/100	774970				
25	25					1/100	42910				
26	20					1/100	221430				

27	25					1/100	4000				
28	25					1/100	7165				
29	27					undiluted	14.59				
30	19					1/10	745.10				
31	22					1/100	16600				
32	23					1/100	306740				
33	22					1/100	7835				
34	22					1/100	6420				
35	28					1/10	83.47				
36	27					1/10	239.60				
37	25.35					1/10	190.70				
38	25					1/100	2128				
39	23					1/100	282650				
40	25					1/100	10870				
41		33				1/10		226.10			
42		21				1/100		129420			
43		24				1/100		6471			
44		25				1/100		13330			
45		26				1/100		8104			
46		39				undiluted		0.39			
47		23				1/100		559970			
48		27				1/10		1084			
49		25				1/100		8901			
50		29				1/10		432.80			
51		18				1/100		545250			
52		31				1/10		203.10			
53		21.33				1/100		408860			
54		27				1/100		3395			
55		24.11				1/100		105390			
56		25.4				1/100		10680			

57		27				1/100		6183			
58		30				1/100		2785			
59			20.6			1/100			9938		
60			21.2			1/100			11880		
61			24.8			undiluted			335.90		
62			42			undiluted			2.61		
63			24.2			1/100			465.70		
64			20.1			1/100			33220		
65			31			1/100			93.80		
66			17			1/100			10640		
67			19			1/100			149080		
68			23			1/100			8151		
69			37			1/100			21.30		
70			27			1/100			1938		
71			27			1/10			581		
72			20			1/100			156920		
73			27			1/100			37860		
74			34			1/10			144.50		
75			31			1/10			92.88		
76			27.6			1/100			11420		
77			23			1/100			31040		
78			24			1/10			1455		
79			21			1/100			576520		
80			24			1/100			13370		
81			19.2			1/100			520460		
82			25			1/100			21870		
83			26			1/100			15460		
84			24			1/100			42820		
85				14.76		1/100				554110	
86				18.97		1/100				93710	

87				19		1/100				41990	
88				21		1/100				281090	
89				25		1/10				138.50	
90				20		1/100				7304	
91				26		1/10				370.60	
92				18		1/100				84400	
93				16.57		1/100				81080	
94				20		1/100				6184	
95				11		1/100				1297130	
96				19		1/100				36850	
97				18		1/100				155000	
98				27		1/10				439.40	
99				20		1/100				21430	
100				17		1/100				469160	
101				20		1/100				19470	
102				34		1/10				2.16	
103				21		1/100				9485	
104				26		1/100				1210	
105	23*		30			1/100	2571*		26800		
106	39.5*		20.9			1/10	3681*		68214		
107	36.6*		22.5			1/10	1350*		19600		
108	24.7*		undetermined			1/10	563.1*		10.38		
109	23.15*		24.4			1/100	2228*		4021		
110	23.6*		25.2			1/100	1211*		704		
111			27.7	34		1/100			4128	5.50	
112					-	1/100					0
113					-	1/100					0
114					-	1/100					0
115					-	1/100					0
116					-	1/100					0

117					-	1/100					0
118					-	1/100					0
119					-	1/100					0
120					-	1/100					0
121					-	1/100					0

Supplementary Table S2: Expected and actual positive and negative samples for a cut-off of 4,000 viral copies/ul

		Observed	
		+	-
**Expected**	+	56	8
	-	5	33

Supplementary Table S3: Expected and actual positive and negative samples for a cut-off of 239.6 viral copies/ul

		Observed	
		+	-
**Expected**	+	21	1
	-	4	15

Figure S1: *dPCR versus Real-Time RT-PCR for Influenza B samples stratified by Ct values.*

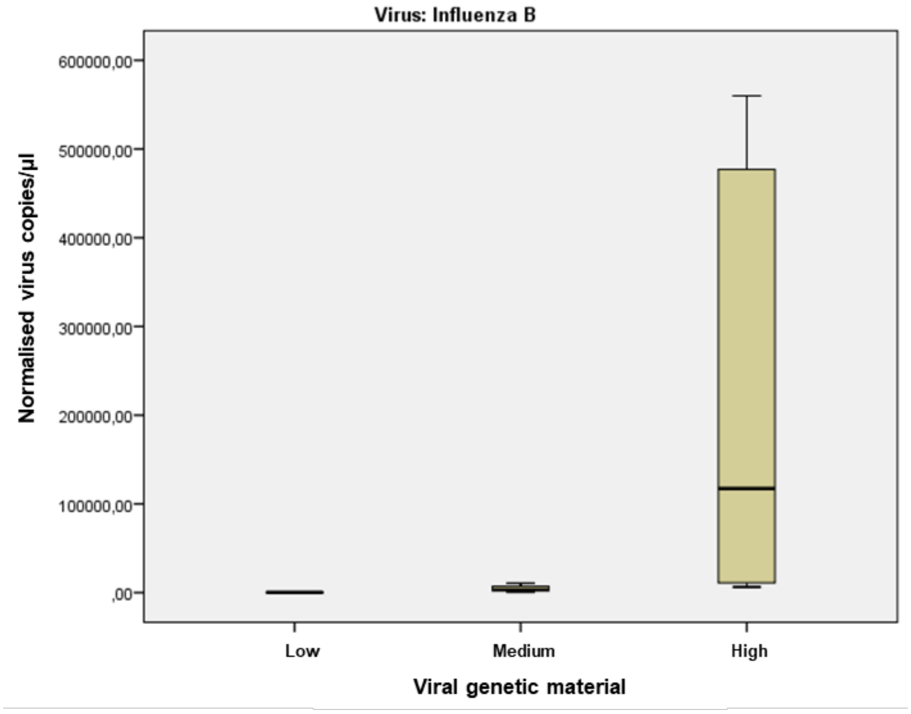

Figure S2: *Detailed distribution of Ct values for SARS-CoV-2 samples.*

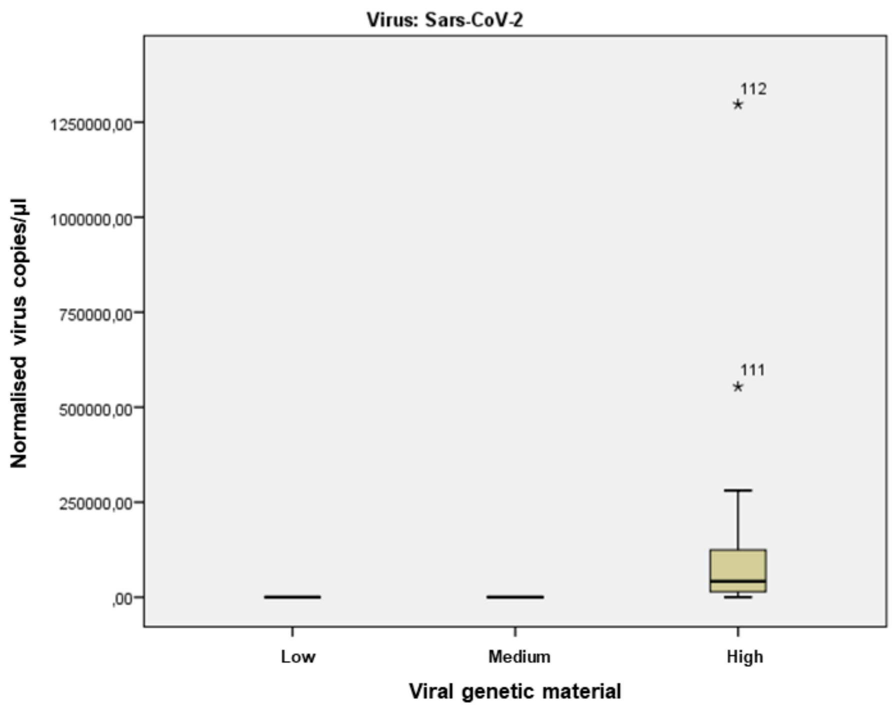

Supplement: Supplementary file 1 [file viruses-17-01259-s001.zip › viruses-3833650-supplementary.pdf]
